# Histological Analysis of Intracranial Cerebral Arteries for Elastin Thickness, Wall Thickness, and Vessel Diameters: An Atlas for Computational Modeling and a Proposed Predictive Multivariable Model of Elastin Thickness

**DOI:** 10.3390/jcm14124320

**Published:** 2025-06-17

**Authors:** Nishanth Thiyagarajah, Alex Witek, Mark Davison, Robert Butler, Ahmet Erdemir, John Tsiang, Mohammed Shazam Hussain, Richard Prayson, Mark Bain, Nina Z. Moore

**Affiliations:** 1 Cerebrovascular Center, Cleveland Clinic Foundation, 9500 Euclid Avenue, S80, Cleveland, OH 44195, USA; thiyagn2@ccf.org (N.T.);; 2 Quantitative Health Sciences, Lerner Research Institute, Cleveland Clinic Foundation, Cleveland, OH 44106, USA; 3 Department of Biomedical Engineering, Lerner Research Institute, Cleveland Clinic Foundation, Cleveland, OH 44106, USA; 4 Department of Pathology, Neuropathology Section, Cleveland Clinic Foundation, Cleveland, OH 44106, USA

**Keywords:** cerebrovasculature, posterior circulation aneurysms, arteriovenous malformations, elastin, computational fluid dynamics, fluid–structure interaction

## Abstract

**Background/Objectives:** Fluid dynamic models of the cerebral vasculature are being developed to evaluate intracranial vascular pathology. Fluid–structure interaction modeling provides an opportunity for more accurate simulation of vascular pathology by modelling the vessel wall itself in conjunction with the fluid forces. Accuracy of these models is heavily dependent on the parameters used. Of those studied, elastin has been considered a key component used in aortic and common carotid artery modeling. We studied elastin thickness to determine if there was significant variation between cerebral artery territories to suggest its importance in cerebral blood vessel biomechanical response and provide reference data for modeling intracranial elastin. Elastin thickness was compared to vessel location, thickness, diameter, and laterality within human intracranial arteries. **Methods:** Tissue was taken from five human cadaveric heads preserved in formaldehyde from each intracranial vessel distribution bilaterally and stained with Van Gieson stain for elastin. A total of 160 normal cerebral vascular artery specimens were obtained from 17 different cerebrovascular regions. Two reviewers measured elastin thickness for each sample at five different locations per sample using Aperio ImageScope (Leica Biosystems, Deer Park, IL, USA). Statistical analysis of the samples was performed using mixed-models repeated measures regression methods. **Results:** There was a significant difference between anterior circulation (6.01 µm) and posterior circulation (4.4 µm) vessel elastin thickness (*p*-value < 0.05). Additionally, two predictive models of elastin thickness were presented, utilizing a combination of anterior versus posterior circulation, vessel diameter, and vessel wall thickness, which demonstrated significance for prediction with anterior versus posterior combined with vessel diameter and wall thickness. **Conclusions:** Elastin thicknesses are significantly different between anterior and posterior circulation vessels, which may explain the differences seen in aneurysm rupture risk for anterior versus posterior circulation aneurysms. Additionally, we propose two potential models for predicting elastin thickness based on vessel location, vessel diameter, and vessel wall thickness, all of which can be obtained using preoperative imaging techniques. These findings suggest that elastin plays an important role in cerebral vascular wall integrity, and this data will further enable fluid–structure interaction modeling parameters to be more precise in an effort to provide predictive modeling for cerebrovascular pathology.

## 1. Introduction

### Cerebrovascular Pathology and Management

Cerebrovascular pathologies effect around 5 million people per year globally and include ischemic stroke, intracranial steno-occlusive disease, cerebral aneurysms (CAs) and cerebral arteriovenous malformations (cAVMs), among others [1,2,3]. For these pathologies patient specific treatment strategies are limited by incomplete characterization of the natural history of lesions; particularly, natural history can be altered by unique variations in individual anatomy, morphology, and clinical history. For CAs and cAVMs, large-scale retrospective and prospective studies have been used to generate predictive scales for rupture risk [2,3,4]. Cerebral aneurysms are relatively common, with an estimated prevalence of 3–5% in the general population [1,5]. Of these patients, many go undiagnosed either until rupture or post-mortem, but a number of these are diagnosed as incidental findings [1,6]. For cAVMs, which carry a 2–3% risk of rupture per year on average, clinical decision-making in the treatment of un-ruptured cAVMs is being questioned after the recent and controversial ARUBA trial comparison of medical management to intervention [1,2,3,4,5,6,7,8,9]. Of course, any medical or surgical management options carry with them the potential for significant procedural morbidity as well. These factors further underscore the need for assistive decision-making tools in pre-procedural clinical management.

Clinically, there are a number of established and emerging neuroimaging utilities such as arterial spin labeling(ASL), high-resolution vessel wall imaging (HR MRI) and endovascular optical coherence tomography (OCT) [10,11,12,13]. Additionally, to help inform a more patient-specific treatment regimen, there has been a recent move to utilize engineering tools typically seen in the aerospace and automotive industries to create a predictive model of cerebrovascular disease progression [14]. This is represented by fluid structure interaction (FSI) models, enabling an evaluation of the combined interactions of a vessel wall and the fluid forces of blood within. Attempts to collect data informing such models have focused mainly on large-scale retrospective natural history studies, which do not include important biomechanical properties data [1,2,3,4,5,6,7,8,9,14]. In order to create FSI models with optimal accuracy, accurate biomechanical parameters need to be incorporated for both the vessel wall and the fluids moving inside it. A better understanding of the cerebral artery and vein histological composition of the vessel wall, including important structural components of elastin and smooth muscle, is needed to begin to understand how the cerebral blood vessel specifically will respond to fluid flow and intra- or extra-vascular-device-induced stress [14,15].

Elastin has long been considered a key layer in the vessel wall to maintain structural integrity. Elastin’s quantity and continuity play a large role in determining both the passive mechanical properties and resulting dynamic behavior of the vessels during a regular cardiac cycle for the aorta and carotid artery. It represents the most abundant protein band in both the internal elastic lamina (IEL) and the external elastic lamina (EEL) [16,17]. However, for cerebral vessels, differing from the systemic vasculature, there is only one layer of elastic lamina without an EEL [18,19]. This thin IEL layer has not been studied extensively in terms of mechanical properties and viscoelastic behavior. Given these structural discrepancies between the large extracranial and cerebral blood vessels, the mechanical properties of the cerebral vasculature cannot necessarily be accurately extrapolated from studies of systemic circulation biomechanics without further functional understanding of this specialized subpopulation of blood vessels themselves [15].There remain many unanswered questions about the passive mechanical properties and dynamic behavior of cerebrovascular tissue requiring further work to be done regarding this area of study [20,21,22].

Additionally, absence or aberrations in elastin content are felt to contribute to both aneurysm formation and eventual weakness leading to rupture, and its thickness in certain areas of the circulation may play a role in the risk of rupture predicted by previous studies [23]. This study focuses on determining the thickness of the internal elastic lamina throughout each branch of the intracranial circulation from samples of cadaver tissues to first determine if there are noticeable interregional differences. The goal of this work in combination with future studies, is to be able to assign a thickness parameter for mechanical testing models by accounting for inter-regional differences in anatomy. This study characterized the initial parameters of the cerebrovascular vessels that can serve as a framework for future models employing the mechanical properties of the vessel wall. 

## 2. Methods

### 2.1. Tissue Procurement and Preparation

Intracranial artery tissue was obtained from 5 formaldehyde-preserved cadaver heads (Innoved Institute, LLC, Elk Grove Village, IL, USA) that were carefully dissected by two trained cerebrovascular neurosurgeons (NZM, AW) in the Cleveland Clinic Foundation Skull Base Dissection Laboratory. Institutional policy was followed carefully in planning the experiments, and use of the specimens did not require institutional board review, as their ethical acquisition from deidentified, non-living subjects delineated non-clinical research per internal policy. A total of 160 normal cerebral vascular artery specimens were obtained from 17 different cerebrovascular regions. Demographics for the 5 cadaver heads are listed in Table 1, with selection criteria for the specimen limited to no known intracranial pathology affecting blood vessels.

Specimens were obtained from each specified location within the vascular territories: internal cerebral artery (ICA), anterior cerebral artery (A1, A2, A3), middle cerebral artery (MCA) (M1, M2, M3, M4), posterior cerebral artery (P1, P2), superior cerebellar artery, anterior inferior cerebellar artery (AICA), posterior inferior cerebellar artery (PICA), vertebral, basilar, and anterior communicating (ACOM) and Posterior Communicating (PCOM) vessels, as summarized in Table 2. Tissue was immediately stored in 10% formalin specimen containers.

### 2.2. Processing and Histological Analysis

**Figure 1:**
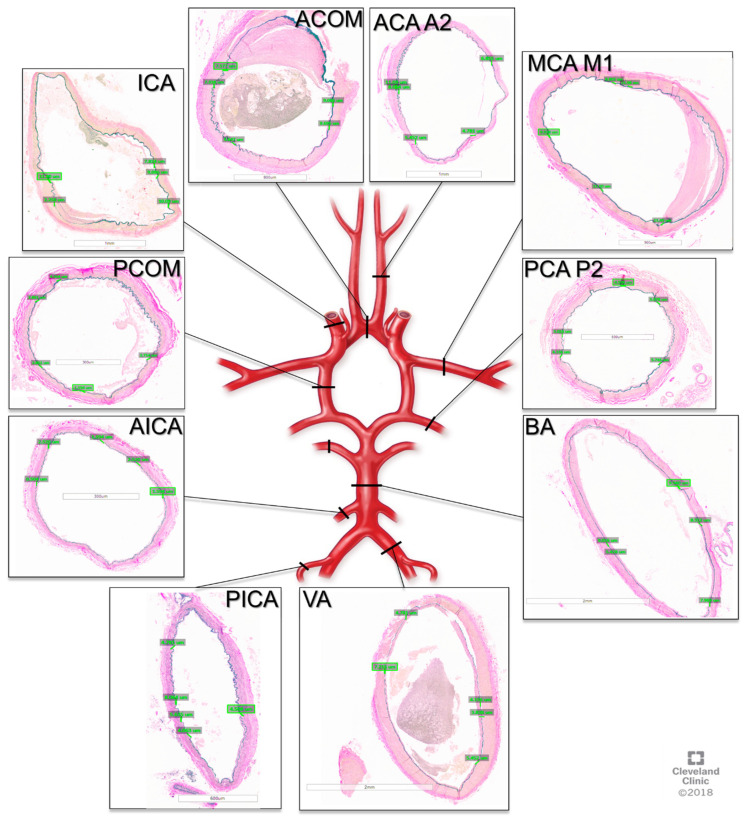
Cross-sections of cerebral arteries around the circle of Willis with Van Gieson staining for elastin. ACOM: anterior communication artery. ACA A2: anterior cerebral artery A2 segment, MCA M1: middle cerebral artery M1 segment, PCA P2, posterior cerebral artery P2 segment, BA: basilar artery, VA: vertebral artery, PICA: posterior inferior cerebellar artery, AICA: anterior inferior cerebellar artery, PCOM: posterior communicating artery, ICA: internal carotid artery.

The tissue was then processed by the Cleveland Clinic Imaging Core Histology Laboratory into paraffin embedded sections and stained with Van Gieson’s stain. Microscope imaging of the slides was then performed, and elastin measurements were obtained using the Aperio Image Scope software v12.3.3 (Leica Biosystems, Deer Park, IL, USA). The measurements were performed by two neurosurgeons (NZM, AW) trained by a neuropathologist (RP) on visualizing elastin, and 5 measurements were taken per sample per person, as depicted in Figure 1.

### 2.3. Statistical Analysis

**Figure 2:**
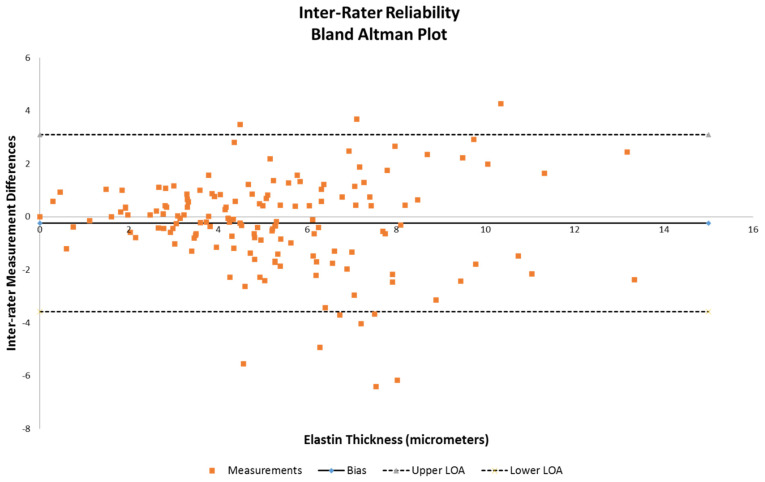
Bland–Altman plot to assess inter-rater reliability between reviewer 1 and reviewer 2 on elastin thickness data.

Analysis was performed on the obtained measures of elastin thickness and vessel wall thickness (defined as intima through adventitia for one side of a vessel diameter of obtained samples that were intact). Also taken into account were the vessel diameter, anterior vs. posterior, and right vs. left lateralities as class variables to examine whether these affected the measured differences in elastin thickness. Inter-rater reliability was evaluated for bias and is demonstrated using a Bland–Altman plot (Figure 2). Multivariable analysis using a mixed-models repeated measures regression method was then performed on the dataset to examine the statistical significance of the anterior/posterior differences against combinations of vessel diameter, wall thickness, and elastin thickness. Specifically, comparisons of (1) elastin thickness versus vessel diameter and anterior/posterior circulation, (2) elastin versus vessel wall thickness and anterior/posterior circulation, (3) vessel wall thickness versus diameter of vessel and anterior/posterior circulation, and (4) elastin thickness alone and anterior/posterior circulation were all tested, with significance being defined as a *p* value < 0.05. All analyses were performed using SAS version 9.4 by biostatistician author RB.

## 3. Results

There was a significant relationship between vessel diameter and elastin thickness as well as between laterality and elastin thickness, with it being greater for the left than the right (Table 3).

There was a significant difference in elastin thickness in anterior versus posterior circulation, with the mean anterior elastin thickness being significantly greater than the posterior elastin thickness. There was a significant difference between anterior circulation (6.1 µm) and posterior circulation (4.4 µm) vessel elastin thickness (*p*-value = 0.0011), as can be seen in Table 4. Additionally, there was a significant association between elastin thickness and wall thickness, with each unit increase in wall thickness seeing an increase in elastin thickness of 0.015 µm (Table 4).

It was also demonstrated that wall thickness was significantly associated with vessel diameter and location (Table 5). Without controlling for other factors, there was a significant association between elastin thickness and anterior and posterior circulation locations (Table 6).

Overall, it was shown that posterior circulation thicknesses (4.45 µm) were significantly lower than anterior circulation elastin thicknesses (5.91 µm) (*p* = 0.044). In an effort to see if a prediction of elastin thickness could be obtained from the other measurements, a co-linearity check demonstrated that elastin thickness can potentially be determined using two subsets of the variables for a predictive multivariable model by combining anterior and posterior location with vessel diameter and wall thickness (Table 7).

**Figure 3:**
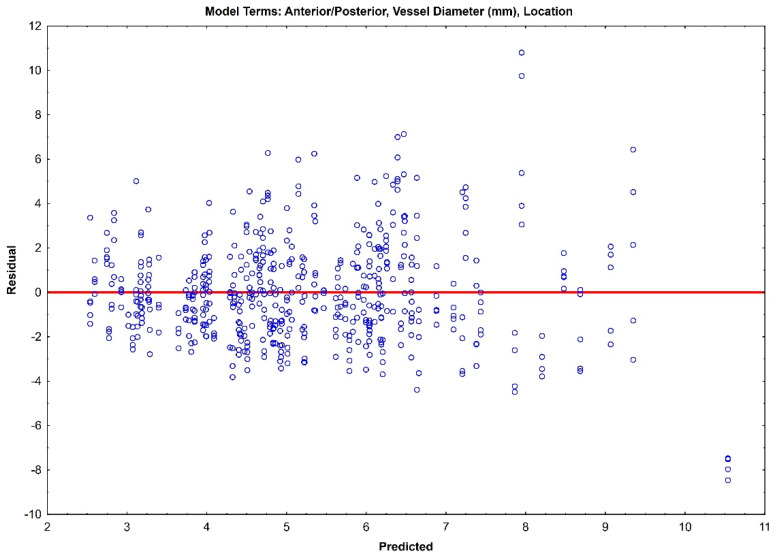
Residual plot for Model 1 with prediction error of ±2.31.

**Figure 4:**
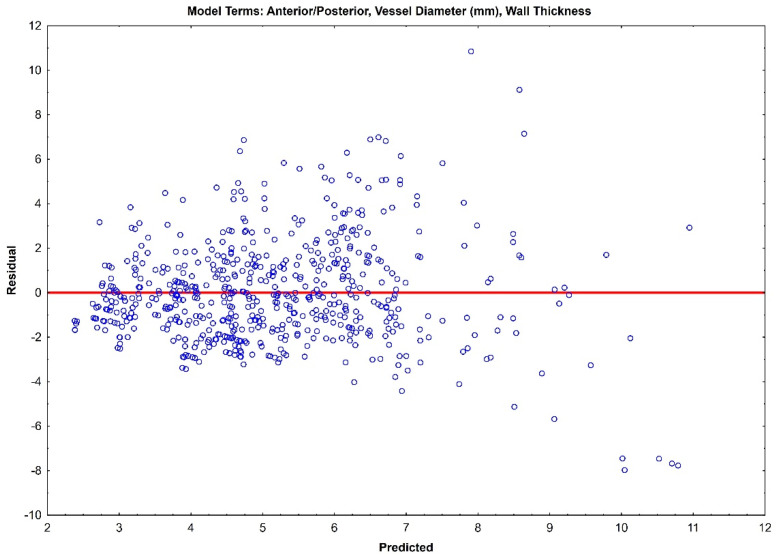
Residual plot for Model 1 with prediction error of ±2.32.

The residual plots of both Model 1 (Figure 3) and Model 2 (Figure 4) do not indicate a real difference between the capabilities of the two models, with a comparable prediction error of ±2.31 and ±2.32, respectively. Given the preliminary nature of this data, both of these models should be retained, and their prediction capabilities should be tested with future data.

Inter-rater reliability demonstrated that the majority of the measurements were within the acceptable range of variance (Figure 2), as indicated by the limits of agreement lines of the Bland–Altman plot, with only 10 of the 160 samples being outside.

## 4. Discussion

This study is a preliminary effort to characterize the intracranial blood vessels in terms of elastin thickness and to show any regional differences that may exist, thus providing a basis for future studies and more accurate structural modeling data for cerebral arteries. From a small sample, we showed a significant difference in the relative thickness of elastin between the anterior and posterior circulation. Additionally, this data shows that the laterality of right versus left also potentially affects elastin thickness. If studied in larger cohorts, this has the potential for correlation with functional differences clinically. For example, further implications of handedness and hemispheric speech dominance may play a role in elastin thickness measurement differences.

### 4.1. Elastin Thickness, Anterior Versus Posterior Circulation, and the Link to Cerebral Aneurysm Rupture Risk

Our findings regarding the significant difference in elastin thickness between anterior and posterior circulation, with the posterior circulation having a thinner elastin wall layer, support prior studies that have looked specifically at the higher incidence of aneurysmal rupture in the posterior circulation. As seen in the International Study of Un-ruptured Intracranial Aneurysms, this suggests that elastin thickness plays an important role in the structural integrity of cerebral vessels [4,15,16,23,24]. This finding may point to a reason for anterior communicating aneurysms within the anterior circulation having a higher rupture risk, similar to posterior circulation aneurysms as compared to other anterior circulation territories where the etiology of this increased relative risk remains unclear and warrants further investigation [1,3,4,5,6,7,8,9]. This study also supports the recent finding that flow-related CAs associated with cAVM occur at higher rates with respect to their embryological derivatives within the posterior infratentorial circulation compared to the anterior circulation [23]. 

### 4.2. Clinical Translation and Predictive Modeling

We have demonstrated two potential predictive models of elastin thickness that may be generated by utilizing anterior versus posterior circulation, vessel diameter, and vessel wall thickness. With larger sample sizes, we will continue to validate which of the two models will have the necessary fidelity to determine elastin thickness. We are actively pursuing determining the viscoelastic properties of the cerebral arteries and veins with comparison to elastin thickness, vessel diameter, location, and vessel wall thickness to further provide structural testing data for cerebrovascular circulation structural modeling. All of the factors included in the predictive models of elastin thickness can soon be obtained from preoperative imaging techniques in the form of cerebral angiography and intravascular optical coherence tomography, making this model directly applicable to individualized patient care. The ultimate goal will be to utilize patient personalized measurements obtained from preoperative imaging to create natural history and treatment predictive models to help guide clinical care.

## 5. Study Limitations

The limitations of our study include our use of a small sample size of patients with a lower standardization of underlying comorbidities, some of which, including age, may have affected the experimental measures collected. However, this was intended as a pilot study to demonstrate a clear difference and the viability of modeling on this basis for future, larger-sample-size cohorts. In addition, a prior study by Fonck et al. indicated that the quantitative amount of elastin found in cerebral arteries remained relatively stable with age [25].

## 6. Conclusions

We have identified a significant quantitative difference in the presence of elastin across interarterial cerebral vessel groupings, namely, that of the posterior and anterior circulation. This potentially provides further insight into previously studied phenomena of hemorrhagic strokes as sequelae of aneurysmal development occurring with higher frequency in the posterior circulation [22,25,26,27].

Elastin expression is well established as a particularly key contributing factor to the mechanical behavior of blood vessels, and it thus remains crucial to understanding development and clinical outcomes associated with incidental aneurysm findings. The current study presents a key starting point for further exploration of the mechanical behavior of the cerebral vasculature. Doing so may prove instrumental to future efforts to provide impartial, patient-specific models of intralesional dynamics and guide clinical decision-making accordingly, particularly in combination with emerging modalities of advanced neuroimaging.

## Figures and Tables

**Table 1:** Demographics of human cephalic specimens.

Specimen	Age	Gender	BMI	Past Medical History
1	80	M	21	Parkinson’s disease, Alzheimer’s, prostate cancer in remission
2	91	M	19	Coronary artery disease, Alzheimer’s
3	87	F	27	Coronary artery disease, Mallory–Weiss tear with GI bleed, dementia, hypothyroidism, cardiomyopathy
4	90	F	23	Congestive heart failure, hypertension, chronic obstructive pulmonary disease, Alzheimer’s
5	51	M	23	End-stage spindle cell carcinoma with brain metastases, hypertension, coronary artery disease, 30 pack per year smoker

**Table 2:** Elastin thickness measurements for each cerebral artery vessel distribution.

	Vessel	Reviewer 1			Reviewer 2			Combined Data		
		Mean (µm)	SD (µm)	Median (µm)	Mean (µm)	SD (µm)	Median (µm)	Mean (µm)	SD (µm)	Median (µm)
Anterior Circulation	ICA	6.3	3.4	5.6	8.9	3.7	8.6	7.6	3.8	7.2
	MCA M1	8.2	3.7	7.6	8.9	3.1	8.6	8.6	3.4	8.2
	MCA M2	5.7	2.0	5.0	8.9	2.2	5.3	5.6	2.1	5.1
	MCA M3	3.6	1.3	3.3	3.7	1.8	3.5	3.7	1.6	3.3
	MCA M4	3.1	1.7	2.9	3.1	1.6	3.2	3.1	1.6	3.0
	ACA A1	7.9	3.4	7.3	7.5	2.5	7.6	7.7	2.9	7.4
	ACA A2	6.7	2.4	6.3	6.3	1.8	6.1	6.5	2.1	6.3
	ACOM	6.2	4.6	5.3	6.8	2.4	6.7	6.5	3.7	6.1
Posterior Circulation	VA	4.3	2.0	4.2	4.2	1.7	3.9	4.2	1.8	4.0
	BA	4.2	3.1	4.0	7.1	4.4	8.3	5.6	4.0	4.3
	PICA	3.3	1.5	3.5	3.3	1.8	3.2	3.3	1.7	3.4
	AICA	2.6	2.0	2.3	2.5	2.1	1.5	2.6	1.7	2.2
	SCA	3.9	1.3	4.0	3.7	1.2	3.8	3.8	1.2	3.9
	PCA P1	6.8	3.7	7.1	6.7	3.0	6.6	6.8	3.4	7.0
	PCA P2	5.7	2.1	5.6	5.6	1.9	5.7	5.7	2.0	5.6
	PCOM	4.2	1.5	3.8	4.3	1.5	4.2	4.2	1.4	4.0
	CV	1.1	1.3	0.6	0.9	1.1	0.3	1.0	1.2	0.5

**Table 3:** Elastin thickness (µm) vs. vessel diameter and anterior/posterior. Mean elastin thickness for anterior = 5.56 µm, mean elastin thickness for posterior = 4.55 µm, mean elastin thickness for left location = 5.25 µm, mean elastin thickness for right location = 4.87 µm.

Parameter	Class Values	Coefficient	Std Error	*p*-Value
Intercept		2.32	0.46	0.001
Anterior/Posterior	Anterior = 1, Posterior = 0	1.01	0.61	0.139
Vessel Diameter (mm)		1.27	0.07	**<0.0001**
Location Right/Left	Left = 1 Right = 0	0.38	0.14	0.024
Observer 1 vs. 2	Observer 1 = 1 Observer 2 = 0	−0.043	0.14	0.77

**Table 4:** Elastin thickness (µm) vs. wall thickness and anterior/posterior. Mean elastin thickness for anterior = 6.10 µm, mean elastin thickness for posterior = 4.38 µm.

Parameter	Class Values	Coefficient	Std Error	*p*-Value
Intercept		2.10	0.30	0.0001
Anterior/Posterior	Anterior = 1, Posterior = 0	1.72	0.35	**0.0011**
Wall Thickness (µm)		0.015	0.001	<0.0001
Right/Left Location	Left = 1 Right = 0	−0.027	0.18	0.88

**Table 5:** Vessel wall thickness (µm) vs. vessel diameter (mm) and anterior/posterior. Mean wall thickness for anterior = 123.76 µm, mean wall thickness for posterior = 157.09 µm, mean wall thickness for left location = 146.32 µm, mean wall thickness for right location = 134.53 µm.

Parameter	Class Values	Coefficient	Std Error	*p*-Value
Intercept		29.42	13.38	0.059
Anterior/Posterior	Anterior = 1, Posterior = 0	−33.32	18.23	0.105
Vessel Diameter (mm)		77.59	2.06	<0.0001
Right/Left Location	Left = 1 Right = 0	11.78	4.02	0.017

**Table 6:** Elastin thickness vs. anterior/posterior only. Mean elastin thickness for anterior = 5.91 µm, mean elastin thickness for posterior = 4.45 µm.

Parameter	Class Values	Coefficient	Std Error	*p*-Value
Intercept		4.46	0.43	<0.0001
Anterior/Posterior	Anterior = 1, Posterior = 0	1.45	0.61	**0.044**

**Table 7:** Two models for elastin thickness.

Model #1				
Parameter	Class Values	Coefficient	Std Error	*p*-Value
Intercept		2.1029	0.41	0.0009
Anterior/Posterior	Anterior = 1, Posterior = 0	1.1277	0.53	**0.066**
Vessel Diameter (mm)		1.3548	0.096	**<** **0.0001**
Right/Left Location	Left = 1 Right = 0	0.4237	0.18	**0.048**
**Model #2**				
Intercept		2.1151	0.35	0.0003
Anterior/Posterior	Anterior = 1, Posterior = 0	1.3325	0.45	**0.0182**
Vessel Diameter (mm)		0.9044	0.17	**<** **0.0001**
Wall Thickness (µm)		0.005754	0.002	**0.0017**

## Data Availability

The original contributions presented in the study are included in the article, further inquiries can be directed to the corresponding author/s.
